# From thought to action: On the relevance of including situational cues in thought about intended actions

**DOI:** 10.1371/journal.pone.0264342

**Published:** 2022-02-23

**Authors:** Torsten Martiny-Huenger, Yevhen Damanskyy, Elizabeth J. Parks-Stamm

**Affiliations:** 1 UiT The Arctic University of Norway, Tromsø, Norway; 2 University of Southern Maine, Portland, ME, United States of America; University of Padova, ITALY

## Abstract

Successful everyday self-regulation often hinges on implementing intended responses at a later time–often in specific situations. We address this self-regulation challenge by examining the role of individuals’ thought about intended actions–and specifically whether it does or does not include situational cues. We hypothesized that including situational cues when thinking about intended actions enables stimulus-response learning, thereby increasing the likelihood of implementing the intended actions. Consequently, we pre-registered and found (*N* = 392, age range 18–94) a positive relationship between the self-reported habitual inclusion of situational cues in thought about intended actions and everyday self-regulation success (assessed by self-reported self-efficacy and self-control beliefs). In addition, we provide exploratory evidence that the inclusion of situational cues in thought about intended actions mediates the relationship between conscientiousness and self-regulation success. We discuss the results and the theoretical perspective in relation to how self-control outcomes can be explained by associative learning.

## Introduction

Humans act in a complex physical and social environment that often does not allow them to implement an intended (e.g., “eat an apple”) or requested (e.g., “provide a colleague with information”) response at the moment the desire or request arises. Successful everyday self-regulation (i.e., aligning responses to goals that apply beyond the immediate situation) often hinges on implementing the wanted or requested responses at a later time–often in very specific situations. We need to buy healthy food (intended response) when we are in the grocery store (critical situation). When a colleague requests that we send them information via email, we need to write the e-mail (intended response) the next time we are in front of the office computer (critical situation) on which the required information is stored. As it is unlikely that we can keep such intentions in our active working memory for an extended time, what triggers the intended action (or thoughts about it) at the critical moment?

In the present research, we explore the role of the *format* of spontaneous thought about the intention when the desire or request initially arises. We hypothesized that including relevant situational cues (stimulus: “When I’m back in my office, [. . .]”, “When I pass by a grocery store, [. . .]”) in thought about intended future actions (response: “I need to send an email to X”, “I’ll buy some fruit”) increases the likelihood of successfully implementing the intended responses at a later point in time [1]. This hypothesis is based on an associative-learning perspective: Whereas including situational cues (i.e., a stimulus) in thought about intended future actions (i.e., a response) entails the potential for stimulus-response learning and subsequent stimulus-driven response control (or remembering), thinking about the intended action without an associated situational cue does not link the response to a specific stimulus. Thus, intended actions may be more likely to be triggered or remembered at the critical point in time if previous thought about the intention included the situational cue. In the remaining introduction, we will present a theoretical and empirical justification for that hypothesis before presenting a study that aims to provide a first test of this assumption by measuring individuals’ self-reported likelihood of including situational cues when thinking about intended future actions and self-reported everyday self-regulation success.

## Verbal stimulus-response learning

Associative learning is typically not the first mechanism that comes to mind when thinking about self-regulation (for exceptions see [2–4]). Associative learning in general–and perception-action links in particular–are typically assumed to emerge from a learning history of direct experiences [5–7]. In the previous examples the person may *not* have a systematic learning history of buying fruit at the grocery store or executing the novel request in front of the office computer, as would be required for associations to form and efficiently guide behavior (i.e., habits [7]). It could be argued that the absence of such direct learning experiences–and thus the absence of beneficial habits–is why opportunities are missed and such situations are perceived as challenges.

However, we argue that beneficial stimulus-response associations can form from repetitively linking a situation and a response in thought (see also [1]). Just as repeated stimulus-response co-occurrences of direct experiences forge links between the stimulus and the response [7], the co-occurrence of verbally triggered mental representations of a stimulus and a response should forge similar links (see [8,9] for a proposal on how such verbally established links may be grounded in sensorimotor areas). Empirical evidence that verbally forged links can guide subsequent behavior in a stimulus-driven manner is provided by research on the self-regulation strategy of if-then action planning.

## A self-regulation strategy based on verbal stimulus-response learning

Our present research concerns the spontaneous inclusion of situational cues when thinking about intended future actions. The relevance of situational cues in thoughts about the future is apparent in the *implementation intention* self-regulation strategy [1,10]. In this self-regulation strategy, users are encouraged to plan their intended actions in an if(situation)-then(response) format. Thus, a central aspect of the planning is to strategically link an intended response to an anticipated critical cue that provides a good opportunity to act. Overall, the empirical evidence converges on the idea that this strategic if-then action planning establishes stimulus-response links that facilitate the execution of the intended responses upon perception of the anticipated stimulus [9,11,12].

Most of the research in the area of if-then action planning has experimentally assigned specific if-then plans or instructed participants to generate if-then plans strategically for themselves [10]. Based on this evidence, we explored the idea that the spontaneous inclusion of critical situations in thought about intended actions guides future behavior–potentially by the same associative mechanism. However, rather than focusing on an explicit verbal self-regulation strategy, our proposal includes any spontaneous thought about relevant situational cues–which could consist of images or verbal information–provided there is an overlap of a stimulus and a response in thought.

In sum, people who habitually include thought about situational cues (stimuli) when thinking about intended future actions (responses) are more likely to establish associative links between the stimulus and the response than people who merely attempt to remember the response. Consequently, creating such associative links increases the likelihood that the situational cues trigger the respective associated responses (or thought about the response), even if the person is preoccupied with other thoughts. This stimulus-driven response activation at the critical moment should increase the likelihood of implementing the intended response successfully. Thus, our central hypothesis is that we will find a positive relationship between habitually including situational cues in thought about intended future actions and successful everyday self-regulation.

## The present study

We tested whether there is a relation between the extent to which a person includes goal-relevant situations in thought about intended future actions and everyday self-regulation success. We measured the habitual inclusion of situational cues in thought about intended future actions with an established (behavioral) habit questionnaire [13]. This habit questionnaire has previously been used to assess habitual thought patterns [14] and we adjusted it to thinking about situational cues in thought about intended future actions.

As general self-regulation success is hard to observe, we relied on individuals’ self-reported judgments assessed by two established measures in the self-regulation domain: self-efficacy beliefs [15] and self-control beliefs [16]. The self-efficacy questionnaire (e.g., “It is easy for me to stick to my aims and accomplish my goals”), assesses people’s beliefs of how successful they are in controlling their environment. It is often seen as a predictor of success. However, we use the questionnaire as an outcome that reflects a person’s self-regulation experiences. This use is based on assumptions that measured levels of self-efficacy reflect actual mastery experiences [17] and that these beliefs are distinct from mere positive illusions [15].

Similarly, self-control beliefs are often seen as a capacity that predicts certain life outcomes [16]. However, not unlike the self-efficacy questionnaire, the self-control questionnaire essentially asks participants to judge how well they deal with self-regulation problems in general (e.g., “I have a hard time breaking bad habits,” “I am able to work effectively toward long-term goals”). Consequently, we used both self-reported self-efficacy and self-control as indicators of prior mastery and self-regulation success experiences.

For exploratory purposes, we included a measure of the Big Five personality traits [18]. This inventory includes conscientiousness, a personality trait related to self-regulation (reviewed by [19]). The trait of conscientiousness includes habitually enacting planning behaviors, making lists, setting timelines, and using a calendar (BIC; [20])–cognitive habits that lead to self-regulatory success. Conscientiousness is inconsistently related to if-then action planning [21,22]. Some research indicates that if-then planning outcomes are independent of conscientiousness [21]. Other research suggests that people high in conscientiousness profit less from instructions to use if-then planning [22]. The latter could result from the difficulty in improving on the high level of self-regulation that highly conscientious people already show. Alternatively, it could suggest that highly conscientious people already think about future actions in an if-then format–reflecing another cognitive habit associated with conscientiousness. If so, the inclusion of situational cues when thinking about intended future actions could (partially) mediate the relation between conscientiousness and self-regulation success in general.

The central hypothesis of the present study, however, concerns the relation between habitually including situational cues in thought about intended future actions and everyday self-regulation success. Based on the argumentation provided in the theoretical introduction, we argue that including situational cues in thought about intended future actions establishes associative links between the situation and action (c.f., [1]). Such associative links that overlap with a person’s goals should facilitate goal progress (c.f., [3]). Regarding our operationalization of everyday self-regulation success, perceived goal progress influences a person’s self-regulation competency believes [17] as measured with self-efficacy/control questionnaires. Consequently, we predicted a positive relation between the self-reported likelihood of including situational cues in thought about intended future actions and self-efficacy/control measurements–a higher habitual inclusion of situational cues should predict higher reported self-regulation success ([23]; Appendix A).

## Methods

### Participants and design

Participants from the United Kingdom were recruited by the European-based marketing research company Toluna [24] to participate in an online questionnaire for a small monetary compensation. Ethics approval has been received from the Department of Psychology, UiT The Arctic University of Norway (ref.: 2017/1912). The sample size was set to a minimum of 350 participants. A high participation rate in the first week of data collection resulted in exceeding this number. We deleted the data of participants who indicated they were younger than 18 and removed one participant who only responded to the first 30% of the questions. The final sample contained the complete data of 404 participants (age *M* = 47.09; *SD* = 17.45) with an age range from 18 to 94 years (218 female, 183 male, 1 “other”, 2 “would rather not tell”). Following the pre-registered data cleaning, the full analyzed sample included 392 participants. We additionally implemented a not pre-registered data exclusion criteria that resulted in an “attentive” subsample of 302 participants (see Data Preparation section for details). The study is based on a correlational design with self-efficacy/control beliefs as the primary dependent variables. The primary predictor variable was the self-reported likelihood of including situational cues when thinking about intended future actions. Personality traits were included for exploratory analyses.

### Materials and procedure

The online questionnaire started with an introduction to situational cues in thinking about intended future actions (see Appendix B). This was followed by three questions aimed at further illustrating the difference between intentions that do versus do not include situational cues (as pre-registered, these questions were not analyzed, see Appendix C). Following this introduction, participants completed the cue-thought habit questionnaire, the self-efficacy scale, the self-control scale, and the Big 5 personality trait inventory. Additionally, after the cue-thought habit questionnaire, participants completed the If-Then Planning Scale [25]. This measure was not pre-registered and is not included in present analyses (see S1 File for more information). The study ended with two questions assessing self-reported comprehension of the concepts “intended actions” and “situational cues”, followed by an attention check question that–if following the instructions attentively–was supposed to be left blank (see Appendix H, Question 3). Finally participants were asked about their age, gender, and whether they answered all questions honestly (see Appendix H).

#### Cue-thought habit questionnaire

Participants answered a 9-item version of Verplanken et al.’s [14] questionnaire on mental habits adjusted to cue-thought habits. The introduction to the questionnaire read as follows: “When thinking about future actions, thinking about specific situations where I can do that action is something …” This phrase was followed by different specifications (e.g., “. . . I do frequently,” “. . . I do automatically”; 7-point scale, anchors “Disagree” and “Agree.”; see Appendix D).

#### Self-efficacy belief

Participants completed the English version of the Brief Self-Efficacy Scale [15]. This is a ten-item measure of generalized self-efficacy (e.g., “It is easy for me to stick to my aims and accomplish my goals”; 7-point scale, anchors “Not at all true” and “Exactly true”; see Appendix E).

#### Self-control beliefs

Participants completed the Brief Self-Control Scale [16]. This is a 13-item measure of generalized self-control (e.g., “I am able to work effectively toward long-term goals”; 7-point scale, anchors “Not at all true” and “Exactly true”; see Appendix F).

#### Big five personality traits

We utilized the Mini-IPIP [18] to measure the Big Five factors of personality (extraversion, agreeableness, conscientiousness, neuroticism, and imagination). This is a 20-item measure (4 items for each factor, e.g., “I like order” for conscientiousness; 7-point scale, anchors “Disagree” and “Agree”; see Appendix G). Even though our exploratory analyses were focused on the conscientiousness factor, for transparency reasons we included the full questionnaire with all five personality traits in the statistical analyses.

### Data preparation

There were very few missing values (<0.15% per scale). Thus, the 1 missing value in the cue-thought habit items (0.028%), the 1 missing value in the self-efficacy items (0.025%), the 5 missing values in the self-control items (0.095%), and the 10 missing values in the Mini-IPIP (Big 5) items (0.124%) were replaced with the median calculated from the participant’s remaining responses to the respective (sub)scale.

#### Pre-registered sample

We removed 6 participants (1.49%) who responded with “No” to the statement: “I answered all of the questions honestly.” An outlier analysis (boxplot method; [26]) on the comprehension questions indicated that values of “1” should be considered outliers. 6 participants were identified as outliers on the “intended future actions” comprehension question (*M* = 5.75; *SD* = 1.30; 1–7 range) and 4 participants were identified as outliers on the “situational cue” comprehension question (*M* = 5.74; *SD* = 1.28; 1–7 range). From these 10 outlier values on two scales, 4 participants were identified as outliers on both scales so that 6 participants (1.5%) were removed from the data.

#### Attentive subsample

We considered participants particularly attentive when they correctly skipped the attention check question that they were instructed to skip. Although not pre-registered (as the question was added to the questionnaire after the pre-registration was submitted), we removed participants who did not skip this question (23.0%) and report the analysis results from this attentive subsample (*N* = 302) in addition to the results of the full, pre-registered sample (*N* = 392) for all major analyses.

#### Cronbach alphas

The internal consistency of all scales were acceptably high (Cronbach’s alpha > .619). Thus, we calculated a mean score for each scale per participant (see Table 1).

**Table 1:** Internal consistency and descriptive statistics of the used questionnaires.

Scale	Items	Cronbach’s alpha	*M* (*SD*)
Cue-thought habit	9	.910	4.80 (1.21)
Self-efficacy	10	.943	5.07 (1.12)
Self-control	13	.809	4.21 (0.95)
Conscientiousness	4	.619	4.99 (1.17)
Agreeableness	4	.750	4.95 (1.30)
Extraversion	4	.746	3.62 (1.41)
Imaginativeness	4	.722	4.56 (1.32)
Neuroticism	4	.643	3.84 (1.25)

*Note*. All items were assessed on 7-point Likert scales.

## Results

### Cue-thought habits and self-efficacy/control (pre-registered hypotheses)

In line with our central prediction, Pearson’s correlation coefficients (see Table 2) indicate that cue-thought habits correlate with self-efficacy and self-control beliefs. Higher values on the cue-thought score are associated with higher self-reports of self-efficacy and self-control. A post-hoc power analysis for a small-to-medium effect size (0.2; [27]) with an alpha level of 0.05 and a sample size of 390 (and for the subsample, 300) results in an achieved power of 99% (97%). All relevant observed effect sizes are higher than 0.2 (0.268–0.496), except for the full-sample analysis for self-control (0.111). Thus, we conclude that our sample size was sufficiently powered. The single analysis for self-control that potentially indicated an underpowered sample is an analysis that may have been biased by a methodological issue discussed in the Discussion section.

**Table 2:** Correlation table.

Variable^a^	1	2	3	4	5	6	7
**1. CTH** ^**b**^	–						
**2. Effic.** ^**c**^	0.496** (0.381**)	–					
**3. Cont.** ^**d**^	0.111* (0.268**)	0.202** (0.364**)	–				
**4. Extr.** ^**e**^	**0.169** **	**0.312** **	**0.085**	–			
**5. Agre.** ^**f**^	**0.150** **	**0.176** **	**0.302** **	**0.293v**	–		
**6. Cons.** ^**g**^	**0.228** **	**0.339** **	**0.621** **	**0.063**	**0.374** **	–	
**7. Neur.** ^**h**^	**-0.046**	**-0.379** **	**-0.407** **	**-0.245** **	-0.128*	**-0.294** **	–
**8. Imag.** ^**i**^	**-0.031**	**0.189** **	**0.195** **	**0.299** **	**0.315** **	**0.212** **	**-0.155** **

*Note*. The table shows the correlation coefficients between all scales assessed in the present study.

a Pre-registered sample *n* = 392; for the central variables, the results for the attentive sub-sample *(n* = 301) are added in parentheses.

b Cue-thought habit

c Self-efficacy

d Self-control

e Extraversion

f Agreeableness

g Conscientiousness

h Neuroticism

i Imaginability.

*p < .05.

**p < .01.

### Exploratory inclusion of the big 5 factors

To investigate the effect of the cue-thought habit score beyond the Big 5 personality factors, we conducted two linear regression analyses with cue-thought habit and the Big 5 personality traits as predictors and either self-efficacy or self-control as outcome variable (Table 3). For self-efficacy as the dependent variable, the model explained about 42% of the observed variance. Cue-thought habit remained a significant predictor beyond the variance explained by the Big 5 factors. For self-control as the dependent variable, the model explained about 44% of the observed variance. However, the effects of the cue-thought variable is not as clear. In the pre-registered full sample, cue-thought habit was no longer a significant predictor when including the Big 5 factors. However, in the attentive subsample, cue-thought habit remained a marginally significant predictor for self-control beyond the variance explained by the Big 5 factors.

**Table 3:** Effects of cue-thought habits and Big 5 factors on self-efficacy and self-control.

		std. *b*	*t*	*p*	Lower and upper CI
DV: Self-efficacy					
	Conscientiousness	0.150	3.354	< .001	0.060, 0.229
	Agreeableness	-0.056	-1.262	.208	-0.125, 0.027
	Extraversion	0.149	3.432	< .001	0.051, 0.187
	Imagination	0.102	2.398	0.017	0.016, 0.158
	Neuroticism	-0.270	-6.466	< .001	-0.316, -0.168
	Cue-thought (attentive subsample)	0.436 (0.261)	10.714 (5.642)	< .001 (< .001)	0.331, 0.480 (0.154, 0.320)
DV: Self-control					
	Conscientiousness	0.518	11.789	< .001	0.351, 0.491
	Agreeableness	0.081	1.856	.064	-0.004, 0.122
	Extraversion	-0.038	-0.903	.367	-0.082, 0.030
	Imagination	0.032	0.771	.441	-0.036, 0.082
	Neuroticism	-0.249	-6.087	< .001	-0.250, -0.128
	Cue-thought (attentive subsample)	-0.023 (0.085)	-0.577 (1.842)	.564 (.066)	-0.080, 0.044 (-0.005, 0.142)

*Note*. The values in parentheses are calculated from the attentive subsample (i.e., participants who correctly responded to the attention check). “std.” = standardized.

### Exploratory mediation analysis (conscientiousness)

#### Self-efficacy

**Fig 1:**
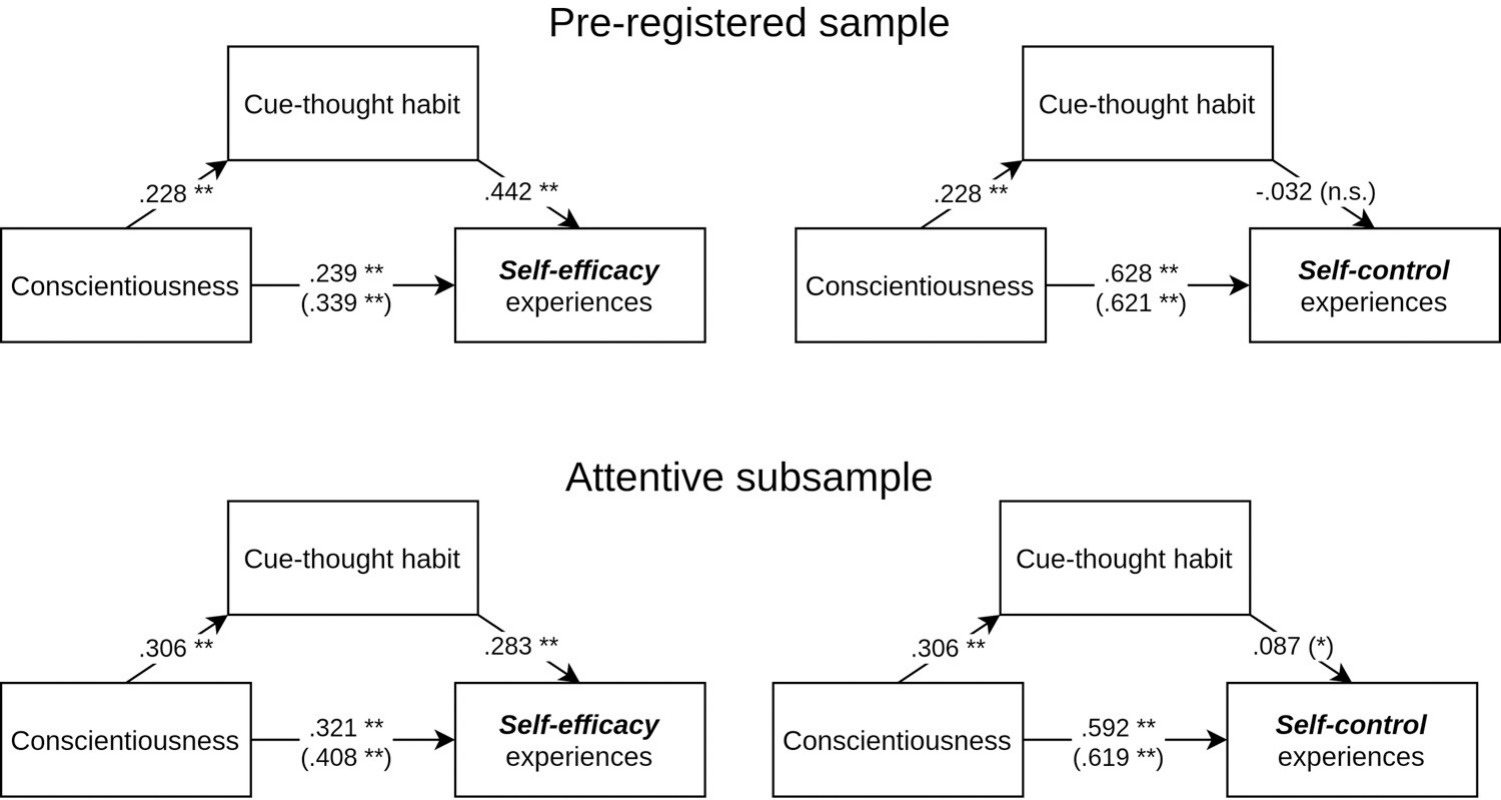
Standardized coefficients for the cue-thought habit mediation between conscientiousness and self-efficacy/control (pre-registered and attentive subsample). Note. ** p < .01, * p < .05, (*) p < .10, n.s. p > .10.

**Fig 2:**
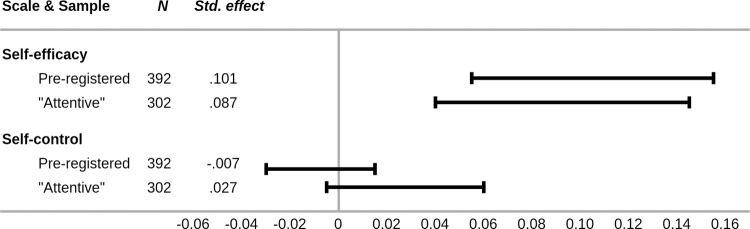
Standardized effect coefficients and confidence intervals for the cue-thought habit mediation effect between conscientiousness and self-efficacy/control (pre-registered and attentive subsample). Note. “Std.” = standardized.

Analyses for both the pre-registered (std. *b* = .101, CI[0.054, 0.154]) and the attentive subsample (std. *b* = .087, CI[0.040, 0.144]) indicated that cue-thought habit is a partial mediator of the relationship between the personality trait of conscientiousness and self-efficacy beliefs (Fig 1, left panel, Fig 2 top panel).

#### Self-control

For self-control as the dependent variable, the results are again more complicated. There is no indication that cue-thought is a mediator of the relationship between conscientiousness and self-control (std. *b* = -.007, CI[-0.030, 0.014]). However, as with all previous analyses, the results from the attentive subsample are more in line with the self-efficacy results. For the attentive subsample, we found a marginally significant mediation effect (std. *b* = .027, CI[-0.005, 0.061]) indicating that there is a tendency for individuals’ cue-thought habit to partially mediate the relationship between conscientiousness and self-control (Fig 1, right panel, Fig 2 bottom panel).

## Discussion

In the introduction, we summarized theoretical and empirical reasons to predict that including situational cues in thought about intended future actions is beneficial for successful everyday self-regulation. In line with this prediction, we found that participants’ self-reported tendency to include situational cues when thinking about intended future actions positively correlated with their self-reported self-efficacy and self-control beliefs.

### Relation to goal specificity and planning in a broad sense

On the broadest level, the inclusion or exclusion of situational cues in thought about intended future actions and their relation to self-regulation success appears to mirror performance effects of goal specificity. Classical goal setting research indicates that more specific and challenging goals lead to higher performance levels [28]. The inclusion of situational cues in thought about intended future actions makes the intention more specific. However, from a goal-setting perspective, specificity alone is not sufficient to increase performance levels [29]. Specificity has benefits in combination with challenging goals (e.g., a specific and challenging goal is superior to a specific and easy goal or a vague and challenging goal). A central assumption as to why specificity has positive performance effects is that a specific goal description provides a concrete anchor and feedback for self-monitoring processes that regulate ongoing behavior [30]. Such self-monitoring mechanisms are different from the mechanism assumed to mediate the positive effects of the inclusion of situational cues in thought about future actions: We hypothesize that the overlap of situation and response in thought leads to associative links that–after being established–make self-monitoring unnecessary. Even when concerned with goal-irrelevant issues, the mere perception of the critical situational cue should trigger the linked response (or thought about the response).

In conclusion, specificity effects in classical goal-setting theory are related to performance outcomes in ongoing tasks (e.g., assembling 50 units of a product in one day). In contrast, our present work is concerned with intended, future tasks that cannot be executed right away. Instead, they have to be initiated at a particular later point in time (for a related discussion of monitoring vs. stimulus-driven mechanisms in prospective memory, [31].

In regard to research on action planning, we narrowed down the broad concept of “planning” to the single aspect of the inclusion of situational cues in thought about intended future actions. This differentiates our study from previous research showing a positive relationship between measurements of planning and goal striving success [32–35]. Although some researchers use terms like “spontaneous implementation intentions” to describe the investigated planning concept in their studies [33,34], we argue these should be considered “planning” in a broader sense. Planning "when, where, and how" to do something often entails specific points in time (e.g., Thursday at 5pm), which leave out the key feature of a *perceivable* situational cue (i.e., visual perception of a certain location or person, internal perceptions of stress, etc.). As we usually do not perceive "Thursday, 5pm" as we would perceive a supermarket or a colleague in the hallway, it is unlikely that such a time specification can act as an effective cue to associatively trigger a response (i.e., if-then planning mechanism; see also [31]). Thus, the present research should be distinguished from this previous research on planning in a broader sense, which could be explained by a range of alternate mechanisms (for a review of such [if-then] planning definition issues see [36]).

In a recent investigation, a measure to assess a person’s planning propensity that is more clearly based on the theory of if-then action planning was developed (If-Then Planning Scale; ITPS; [25]). The researchers found positive relations between the ITPS and goal-achievement in different domains (see Appendix I). However, although the questionnaire includes single items that relate to our present focus (e.g., "I envisage what obstacles could arise"), the questionnaire as a whole goes beyond our present focus on situational cues and their link to responses. For example, some items refer to planning "when and where" to do something, and therefore are likely to be misunderstood by naive participants as plans in a more general sense. Furthermore, the questionnaire contains items that target additional aspects derived from if-then planning research in general (e.g., whether planning is about seizing opportunities versus overcoming obstacles).

In conclusion, the superficial similarity of the If-Then Planning Scale [25] and our idea to measure the habitual inclusion of situational cues triggered its last-minute inclusion in our study (see Method and Appendix I) after our pre-registration was submitted. Their results converge and substantiate our present empirical evidence. However, in contrast to previous studies on self-initiated planning in general [32,33] and the IFPS’s relationship to the if-then planning research agenda in general [25], our present research focuses on a single, very specific aspect of planning: the potential for associative learning when including situational cues in thought about intended future actions. We therefore define and provide some evidence for this specific and clearly defined factor as one mechanism that contributes to the implementation of intended actions.

### Differences in results for self-efficacy and self-control?

We considered both the self-efficacy and self-control questionnaire as reflecting beliefs about everyday self-regulation success; beliefs that are based on prior mastery experiences [17]. On first sight, our present data appears to suggest a difference in the predictive power of cue-thought habits on self-efficacy and self-control. However, methodological differences in the inclusion of reverse-scored items between the different questionnaires may explain this effect. Whereas the self-control questionnaire includes reverse-scored items, the cue-thought habit questionnaire and the self-efficacy questionnaires do not. Less attentive participants who responded similarly to all items (e.g., always the highest or lowest scale option; henceforth referred to as low-variance respondents) would therefore distort the observed relations between the questionnaires. Without reverse-scored items, low-variance respondents can artificially increase positive relationships between questionnaires because the invariant responses will be either high or low for both questionnaires. However, if one of the questionnaires includes reverse-scored items, the invariant high or low responses would reduce the observed correlation because one score would aggregate to an indifferent mid-point score that does not systematically relate to the other invariantly high or low score.

Whereas the pre-registered full sample includes participants with zero variance in their responses (*n* = 13; cue-thought habit, self-efficacy, and self-control questionnaires), no low-variance respondents remained in the attentive subsample. In line with the previous reasoning, the positive relation between cue-thought habit and self-efficacy (i.e., both without reverse-score items) is high in the full sample (24% of the explained variance) and relatively lower in the attentive subsample (14%). We suggest that the relation is overestimated in the full sample, and the attentive subsample’s 14% of explained variance is more reliable. In contrast, the positive relation between cue-thought habit and self-control (which did include reverse-score items) is low in the full sample (1%) and relatively higher in the attentive subsample (7%). Similar to the previous reasoning, we suggest the relation is underestimated in the full sample, and the attentive subsample’s 7% explained variance is more reliable. This reduces the apparent difference between how well cue-thought habits predict self-efficacy and self-control from a 23% difference to a mere 7% difference. Thus, we believe that the results are best (and most conservatively) described as showing a positive relation between cue-thought habits and both self-efficacy and self-control (a similar effect-pattern difference between the full sample and the attentive subsample is evident in the mediation analyses discussed in the Personality Traits and Mediation of Conscientiousness section).

Besides this methodological argument, there are theory-based explanations for the different effects. For example, the self-control items generally target aspects of avoiding or stopping unwanted responses whereas the self-efficacy items are more about engaging with intended behaviors. Our central idea of facilitating behaviors by linking them to critical situations would function for both: thinking of something unwanted and linking it to an alternative response and thinking about a good opportunity and linking an intended behavior. However, most of our examples in the instructions referred to the latter, which may have had more substantial overlap with the content of the self-efficacy items. In conclusion, should a difference between the self-efficacy and self-control questionnaires remain when methodological differences are eliminated, future research is required to validate a theoretical cause of the observed differences.

### Personality traits and mediation of conscientiousness

Conscientiousness has been considered a relevant personality trait in self-regulation research [19] and if-then action planning [21,22]. The inclusion of situational cues in thoughts about intended future actions can be perceived as a more thorough kind of planning than merely attempting to remember the intention. This bears similarities to the behaviors that characterize conscientious people (e.g., making lists and using a calendar or datebook [20]). Thus, we argue that including situational cues in thinking about intended future actions is one additional characteristic of what defines conscientiousness. In line with this argument, in addition to finding evidence for a positive relation between conscientiousness and cue-thought habits, we found evidence that cue-thought habit partly mediates the relationship between conscientiousness and self-efficacy and self-control (albeit weaker for the latter and only in the attentive subsample). Again, as the self-control scale contained reversed items, we believe that the results from the attentive subsample are more reliable. This fits with a general perspective that less conscientious individuals who do not imagine relevant situations to enact planned behaviors may find little success in following through with their intentions. However, highly conscientious individuals who link a situation to an intended action create stimulus-response links that later contribute to the guidance of behavior in line with the individual’s goal. The only other personality trait that consistently predicted both self-efficacy and self-control in our study was neuroticism. Conscientiousness and neuroticism are negatively correlated. Thus, from the present perspective, high neuroticism may analogously undermine the inclusion of situational cues in thought about intended future actions, potentially because of more mental noise [37,38].

### Limitations

The conclusions drawn from the present empirical data are clearly limited by the correlational nature of the study design. While we present a theory-driven causal direction (i.e., cue-thought habits driving self-regulation success), the presented data cannot provide conclusive evidence for this causal path. However, this is a limitation that is somewhat unavoidable. An experimental approach that asks some participants to include situational cues in their thought about future actions and discourages others could not answer our central question of whether the *spontaneous* (i.e., naturally occurring) inclusion of situational cues relates to self-regulation success. Such an experimental approach would shift the research question to the well-investigated topic of the effects of strategically-created if-then plans (reviewed by [10]).

Another limitation is the use of self-reported self-efficacy and self-control beliefs as indicators of everyday self-regulation success. This limitation has two aspects. First, an actual observation of successful or unsuccessful self-regulation behavior would be preferred over the self-report assessment in our present study. Not only are our measurements self-reports that may not accurately reflect the underlying concept, our cue-though habit questionnaire additionally requires metacognitive insight from the participants into their thought processes. To minimize potential misunderstanding, we provided a thorough introduction to the idea of including situational cues in thought about intended actions and a range of easy-to-understand examples (i.e., “dummy” questions that we did not analyze). Whereas a more naturalistic observation would be preferred to the present self-report approach, such a study would require significant resources. The present positive evidence obtained from self-report data clearly indicates that it is worth using these resources to test our hypotheses with more realistic behavioral (outcome) measures.

The second aspect is our use of the self-efficacy and self-control questionnaire as dependent variables reflecting everyday mastery experiences (i.e., self-regulation success). Both questionnaires are often used as predictors for performance in different domains [39]. However, prior empirical evidence and a general interest in self-efficacy/control as predictors is not in conflict with our present use as dependent variables. Bandura [17] listed mastery experiences as the main source of self-efficacy beliefs, and there is empirical evidence that self-efficacy relates more strongly to past performance than future performance [40]. In sum, despite its frequent use as predictor, responses to questions like “It is easy for me to stick to my aims and accomplish my goals” (self-efficacy) and “I am able to work effectively toward long-term goals” (self-control) reflect prior mastery experience and can thus serve as an operationalization of self-regulation success. In the next and final section, we will move away from the specifics of the questionnaires to a broader discussion of the conceptual idea of self-control as an *in situ* act of will.

### Where to look for mechanisms of self-control

Instead of attributing a self-regulation outcome to an *in situ* act of will, we attribute it to prior associative learning based on verbal stimulus-response contingencies. This provides a learning-based alternative to, for example, strength models of self-regulation [41]. Self-control is often defined as “the ability to override or change one’s inner responses” [16] (p. 274). This “ability”, however, is usually not further defined [42,43]. In strength models of self-control, the critical mechanisms needed to override an inner response are typically located at the time the response change is observed. Our present associative-learning perspective, however, does not involve such a *in situ* act of will. We rather assume that a deviation of a person’s behavior from prior routines is driven by continuous prior (associative-)learning experiences. In other words, we assume that behavior change is not a result of an “ability” at work at the time of observing the response change but a result of learning experiences–learning experiences resulting from circumstantial or strategic verbal processing of stimulus-response contingencies (e.g., linking situational cues to intended responses).

This perspective is in line with research showing that beneficial habits—not *in situ* effortful control—are associated with successful self-regulation [2–4]. Our present proposal provides an answer to a critical open question related to this finding: How do (some) people establish these beneficial habits? We argue that one answer is systematic and repetitive thought in a stimulus-response format. The overlap of stimulus and response allows for associative learning from thought, which increases the likelihood of implementing novel behaviors upon contact with the situational cue. The greater likelihood of implementing a novel behavior due to verbal stimulus-response learning potentially increases the probability of further (typical) associative learning from direct experiences to form beneficial habits. In conclusion, our present perspective contributes to illuminating mechanisms of behavior change mainly by arguing not to look for these mechanisms at the salient point in time in which the behavior change is observed, but to examine instead the learning mechanisms at work before the observed change.

## Conclusion

Based on an associative-learning perspective that includes associative learning from direct and indirect stimulus-response contingencies, we hypothesized that there is a positive relation between a person’s habitual inclusion of situational cues in thought about intended future actions and the person’s everyday self-regulation success. We provide evidence for this relationship by measuring everyday self-regulation success via self-reported self-efficacy and self-control questions that reflect everyday mastery experiences. In addition, in an exploratory analysis, we provide evidence that the extent to which a person includes situational cues in thought about intended future actions partially mediates the relationship between conscientiousness and everyday self-regulation success. This evidence indicates that the inclusion of situational cues may be one cognitive habit that characterizes conscientiousness in addition to other previously reported kinds of planning behavior. Finally, the present perspective highlights how self-regulation outcomes can be explained by associative learning.

## Supporting information

S1 File (DOCX)
