# Supplementary material for: From thought to action: On the relevance of including situational cues in thought about intended actions
Source: PLoS One. 2022 Feb 23;17(2):e0264342. doi: 10.1371/journal.pone.0264342 (PMC8865665; doi:10.1371/journal.pone.0264342)
Supplement: S1 File — (DOCX) [file pone.0264342.s001.docx]

Supporting information
A. Preregistration template from AsPredicted.org
Hypothesis
Can we use a habit questionnaire to assess the degree to which an individual thinks in an if-then format when thinking about intended future actions? Is there a relation between habitually thinking in an if-then format and self-efficacy, self-control, and personality-trait measurements? We specifically hypothesize a positive relation between habitual if-then thought and self-efficacy/self-control. A higher degree of habitual if-then thought should relate to higher self-efficacy and self-control. Personality traits are included for exploratory purposes.
Dependent variable
Predictor variable: "Habitual negative thoughts" questionnaire (Verplanken, Friborg, Wang, Trafimow, & Woolf, 2007) adopted to thoughts in an if-then format (excluding items 5, 6, and 10)
Main DVs (self-report scales): self-efficacy (Brief Self-efficacy Scale; Schwarzer, Bäßler, Kwiatek, Schröder, & Zhang, 1997) and self-control (Brief Self Control Measure; Tangney, Baumeister, & Boone, 2004)
Exploratory: Big Five (tiny version; Donnellan, Oswald, Baird, & Lucas, 2006)
All other questions recorded
1) The questionnaire starts with 3 "dummy" questions. Their content is related to if-then like thought but the questions are merely introduced to help the participants understand the relevant concepts before the actual habit questionnaire. [see Appendix B]
2) Two questions at the end ask whether participants believe that they have understood the concepts *future actions* and *thought about future actions that include situations*
3) Only year of birth and gender are assessed as demographic questions
4) Participants are asked whether they responded honestly.
Analyses
- Inter-item correlations within scales and Cronbach alpha to determine internal consistency for each scale, potentially removing problematic items.
- Factor analysis to examine the underlying factor structure.
- Regression model with if-then habit scale as predictor and self-efficacy and self-control as dependent variables.
Outliers and Exclusions
The questions assessing whether participants believe that they have understood the relevant concepts will be checked for outliers and outlier participants towards the lower values (i.e., have not understood the concepts) will be removed (boxplot method). Participants who indicate that they have not honestly responded will be removed.
Sample Size
A commercial company will be hired to recruit 350 participants.


B. Cue-thought questionnaire instructions
In our daily lives we often have to remember to do something in the future (buying milk, answering an email, buying a birthday present for a loved one). We also have to remember general intentions (e.g., to be more healthy) that relate to different actions in the future (e.g., choosing healthy salads when we’re in a restaurant, buying fruits in the grocery store).
Our questionnaire is about such intentions to do something in the future, which we call “future actions.” The remaining questionnaire is about the different ways we think about future actions. In particular, we are interested in the extent to which thoughts about future actions include specific situations where the actions could be performed.
Sometimes thoughts about future actions include specific situations where they could be performed. These situations often represent good opportunities to act or situational cues that can remind us to act, like: “Next time I’m in the shopping center, I’ll look for a birthday present for my friend!”
Whether or not your thoughts about the future are usually linked to situations like this is the focus of this questionnaire. More specifically, we are not interested in thoughts about specific times or dates (e.g., Thursday, 5pm), but rather in environmental cues, that is, something you encounter and perceive (“when I see my friend”, “when I leave work”).

C. Illustrative questions
As you go about your daily activities, you suddenly remember that you have to buy a birthday present for a friend. Which sounds more like your first thought?
“I have to remember to buy a present.” (focus on action alone)
“When I’m in the shopping mall this evening, I will buy a present.” (focus on situation and action)
The doctor tells you to be more physically active for your health. Which sounds more like your first thought?
“Okay, I need to exercise!” (focus on actions alone)
“When and where do I have opportunities to work on this?” (focus on situations and action)
Imagine you run out of toilet paper. Which sounds more like your first thought?
“I should remember to buy toilet paper!” (focus on actions alone)
“After leaving work, I should stop at the store.” (focus on situations and action)

D. Cue-thought habit questionnaire
“The following questions are also about thoughts about future actions that include both situations and actions. How likely is it that your thoughts and plans about future actions include situations where you can perform the actions (e.g., “When I’m in the shopping mall”, “After leaving work”). It might not be easy to make judgements like this about yourself, but please try to imagine how you normally think. Please indicate how much you agree or disagree with the following statements.
When thinking about future actions, thinking about specific situations where I can do that action is something …”
1.  “I do frequently.”
2.  “I do automatically.”
3.  “I do unintentionally.”
4.  “That feels sort of natural to me.”
5.  “I do every day.”
6.  “I start doing before I realize I’m doing it.”
7.  “I would find hard not to do.”
8. “That’s typically ‘me’. ”
9. “I have been doing for a long time.”
As specified in the pre-registration, we removed 3 items from the original 12 item questionnaire that conflicted with our topic of thinking about intended future actions (e.g., “... I don’t do on purpose”). Although these questions are well-suited to describing habitual behaviors, we were concerned that participants would confuse questions about “purpose” and “thinking” with the overall topic of thinking about intended future action – instead of considering it with respect to the inclusion of thinking about situational cues

E. Self-efficacy questionnaire
1. I can always manage to solve difficult problems if I try hard enough.
2. If someone opposes me, I can find means and ways to get what I want.
3. It is easy for me to stick to my aims and accomplish my goals.
4. I am confident that I could deal efficiently with unexpected events.
5. Thanks to my resourcefulness, I know how to handle unforeseen situations.
6. I can solve most problems if I invest the necessary effort.
7. I can remain calm when facing difficulties because I can rely on my coping abilities.
8. When I am confronted with a problem, I can usually find several solutions.
9. If I am in a bind, I can usually think of something to do.
10. No matter what comes my way, I’m usually able to handle it.

F. Self-control questionnaire
1. I am good at resisting temptation.
2. I have a hard time breaking bad habits. (r)
3. I am lazy. (r)
4. I say inappropriate things. (r)
5. I do certain things that are bad for me, if they are fun. (r)
6. I refuse things that are bad for me.
7. I wish I had more self-discipline. (r)
8. People would say that I have iron self- discipline.
9. Pleasure and fun sometimes keep me from getting work done. (r)
10. I have trouble concentrating. (r)
11. I am able to work effectively toward long-term goals.
12. Sometimes I can’t stop myself from doing something, even if I know it is wrong. (r)
13. I often act without thinking through all the alternatives. (r)

G. Personality traits questionnaire
1. I am the life of the party. (extraversion)
2. I sympathize with others’ feelings. (agreeableness)
3. I get chores done right away. (conscientiousness)
4. I have frequent mood swings. (neutroticism)
5. I have a vivid imagination. (imaginability)
6. I don’t talk a lot. (extraversion) (r)
7. I am not interested in other people’s problems. (agreeableness) (r)
8. I often forget to put things back in their proper place. (conscientiousness) (r)
9. I am relaxed most of the time. (neutroticism) (r)
10. I am not interested in abstract ideas. (imaginability) (r)
11. I talk to a lot of different people at parties. (extraversion)
12. I feel others’ emotions. (agreeableness)
13. I like order. (conscientiousness)
14. I get upset easily. (neutroticism)
15. I have difficulty understanding abstract ideas. (imaginability) (r)
16. I keep in the background. (extraversion) (r)
17. I am not really interested in others. (agreeableness) (r)
18. I make a mess of things. (conscientiousness) (r)
19. I seldom feel blue. (neutroticism) (r)
20. I do not have a good imagination. (imaginability)

H. Additional questions
1. I believe I understood what was meant by thinking about *future actions*.
2. I believe I understood what was meant by thinking about future actions in a format that included *situations* in which the action could be performed.
[7-point scale, anchors “Disagree” and “Agree”]
3. Please do not answer this question, leave the scale unmarked and continue with the "Year of birth" question.
	[7-point scale, anchors “Disagree” and “Agree”]
4. Year of birth: [text field]
5. Gender [options: “Female”, “Male”, “Other”, “Would rather not answer”]
6. I answered all of the questions honestly. [options: “Yes”, “No”]


I. If-then planning scale
In the two days between submitting the pre-registration and launching the present study, we were made aware of a manuscript pre-print that appeared to have a similar focus as the present study. Thus, we included the questionnaire used in that article (If-then Planning Scale, ITPS; Bieleke & Keller, 2020) in our study after the cue-thought habit questionnaire. However, a more thorough assessment made us conclude that the questionnaire is not highly relevant to our research question. The ITPS score correlates highly with the cue-thought habit score (r = .617, p < .001), which is not surprising as both assess participants propensity for planning intended actions. However, an exploratory factor analysis (JASP Version 0.14) performed on all items of the cue-thought habit and IFPS questionnaires indicated two factors differentiating the two questionnaires. Factor 1 (cue-thought habit items) explained 30.7% of the variance with factor loadings from .487 to .841. Factor 2 (IFPS) explained 24.4% of the variance with factor loadings from .522 to .884. We discuss the IFPS and its differences to our present aim in the discussion section.
